# Endothelial Cell-Selective Adhesion Molecule Contributes to the Development of Definitive Hematopoiesis in the Fetal Liver

**DOI:** 10.1016/j.stemcr.2019.11.002

**Published:** 2019-12-05

**Authors:** Tomoaki Ueda, Takafumi Yokota, Daisuke Okuzaki, Yoshihiro Uno, Tomoji Mashimo, Yoshiaki Kubota, Takao Sudo, Tomohiko Ishibashi, Yasuhiro Shingai, Yukiko Doi, Takayuki Ozawa, Ritsuko Nakai, Akira Tanimura, Michiko Ichii, Sachiko Ezoe, Hirohiko Shibayama, Kenji Oritani, Yuzuru Kanakura

**Affiliations:** 1 Department of Hematology and Oncology, Osaka University Graduate School of Medicine, Suita 565-0871, Japan; 2 Genome Information Research Center, Research Institute for Microbial Disease, Osaka University, Suita 565-0871, Japan; 3 The Institute of Experimental Animal Sciences Department of Medicine, Osaka University, Suita 565-0871, Japan; 4 Department of Anatomy, Keio University School of Medicine, Shinjuku-ku, Tokyo 160-8582, Japan; 5 Department of Immunology and Cell Biology, Osaka University Graduate School of Medicine, Suita 565-0871, Japan; 6 Department of Vascular Physiology, National Cerebral and Cardiovascular Center Research Institute, Suita 564-8565, Japan; 7 Department of Hematology, Graduate School of Medical Science, International University of Health and Welfare, Narita 286-8686, Japan

**Keywords:** definitive hematopoiesis, endothelial cell-selective adhesion molecule, hematopoietic stem cells, endothelial cells, fetal liver

## Abstract

Endothelial cell-selective adhesion molecule (ESAM) is a lifelong marker of hematopoietic stem cells (HSCs). Although we previously elucidated the functional importance of ESAM in HSCs in stress-induced hematopoiesis in adults, it is unclear how ESAM affects hematopoietic development during fetal life. To address this issue, we analyzed fetuses from conventional or conditional ESAM-knockout mice. Approximately half of ESAM-null fetuses died after mid-gestation due to anemia. RNA sequencing analyses revealed downregulation of adult-type globins and Alas2, a heme biosynthesis enzyme, in ESAM-null fetal livers. These abnormalities were attributed to malfunction of ESAM-null HSCs, which was demonstrated in culture and transplantation experiments. Although crosslinking ESAM directly influenced gene transcription in HSCs, observations in conditional ESAM-knockout fetuses revealed the critical involvement of ESAM expressed in endothelial cells in fetal lethality. Thus, we showed that ESAM had important roles in developing definitive hematopoiesis. Furthermore, we unveiled the importance of endothelial ESAM in this process.

## Highlights

• ESAM deficiency causes life-threatening anemia in fetuses
• ESAM deficiency impairs adult-type hemoglobin synthesis
• ESAM deficiency downregulates expression of *Alas2* and *adult-type globins* in HSCs
• Endothelial ESAM plays roles in the ontogeny of definitive hematopoiesis

## Graphical Abstract

**Figure:**
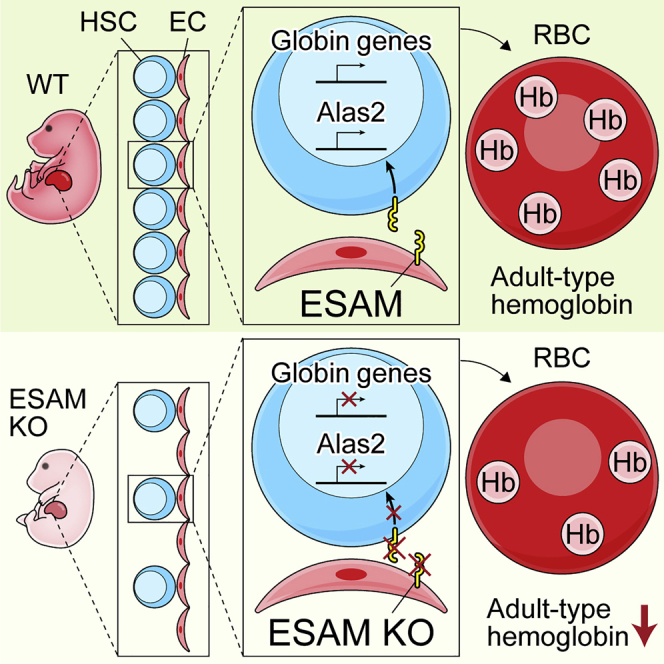

## Teaser

Ueda et al. shed light on the contribution of endothelial cell-selective adhesion molecule (ESAM) to the ontogeny of definitive hematopoiesis. ESAM deficiency caused high mortality in fetuses after mid-gestation. Expression of *Alas2* and *adult-type globin* genes decreased in ESAM-null HSCs, which resulted in the impairment of development of adult-type erythropoiesis. The authors also revealed functional involvement of ESAM-expressing endothelial cells.

## Introduction

Lifelong blood production depends on hematopoietic stem cells (HSCs) and their immediate progeny (Busch et al., 2015, Sun et al., 2014). Originating in the wall of the developing aorta and arteries, HSCs contribute to “definitive” hematopoiesis arising in mid-gestation embryos. These HSCs are considered authentic because they are endowed with the potential for B and T lymphopoiesis as well as adult-type erythropoiesis (Cumano et al., 1996, McGrath and Palis, 2008, Medvinsky and Dzierzak, 1996, Yokota et al., 2006).

Because HSCs emerge in close association with vessel endothelial cells (ECs), which are referred to as the “hemogenic endothelium” (Bertrand et al., 2010, Boisset et al., 2010, Dzierzak and Speck, 2008, Kissa and Herbomel, 2010), a number of endothelial-related antigens are expressed on developing HSCs. Such endothelial antigens include Tie2/angiopoietin receptor-2, CD144/vascular-endothelial (VE) cadherin, CD31/platelet endothelial cell adhesion molecule-1 (PECAM-1), CD105/endoglin, and CD34 (Chambers et al., 2007, Cho et al., 2001, Nishikawa et al., 1998, Takakura et al., 1998, Yoder et al., 1997). These antigens are useful for identification of developing HSCs among embryonic tissues; however, most are downregulated as the hematopoietic system matures into the adult-type system (Mikkola and Orkin, 2006, Yokota et al., 2012). In addition, the functional significance of these antigens in the development of hematopoiesis has remained largely unknown.

We previously reported that endothelial cell-selective adhesion molecule (ESAM), which was initially identified as an EC-specific antigen, serves as an effective marker for lifelong HSCs in mice and humans (Hirata et al., 2001, Ishibashi et al., 2016, Yokota et al., 2009). Before the establishment of the first definitive HSCs, ESAM is highly expressed on the hemogenic endothelium in the developing aorta (Yokota et al., 2009). We also observed that ESAM expression levels differ substantially between myeloid-erythroid progenitors in the yolk sac and definitive HSCs in the intra-embryonic sites (Yokota et al., 2009).

ESAM expression in HSCs is functionally important for hematopoiesis in the adult bone marrow (BM). ESAM expression levels reflect activation of HSCs after administration of 5-fluorouracil (5-FU) (Sudo et al., 2012). Furthermore, ESAM deficiency causes life-threatening myelosuppression, particularly severe anemia, in stress-induced hematopoiesis (Sudo et al., 2016). These results highlighted the role of ESAM in developing hematopoiesis of embryos and/or fetuses, during which rapid, substantial production of blood cells is required.

Thus, in the current study, we analyzed how ESAM expression influenced hematopoietic development during fetal life using ESAM-knockout (KO) and conditional ESAM-knockout (cKO) mice. As a result, we found that ESAM had unique functions in the ontogeny of definitive hematopoiesis, particularly in the development of adult-type erythropoiesis. Our findings provide important insights into the development of hematopoiesis in fetuses because ESAM is a rare endothelial-related antigen shown to be functionally involved in the development of definitive HSCs.

## Results

### ESAM Deficiency Reduced Birthrates in Mice

**Figure 1:**
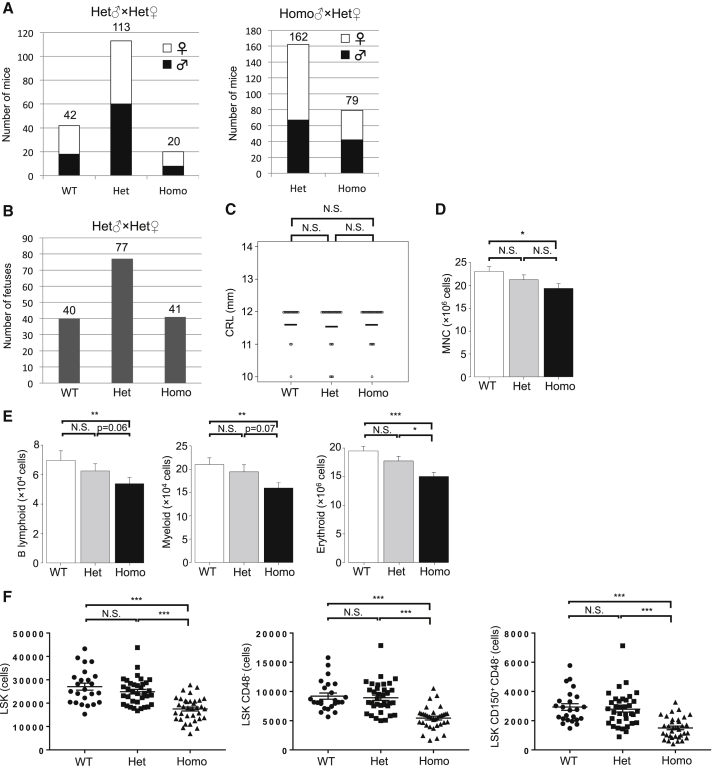
ESAM Deficiency Reduced Birthrates and Disrupted Hematological Development in the Fetal Liver (A) The number of newborn ESAM-KO mice generated from the mating of ESAM heterozygous-KO (Het) males and ESAM Het females (left) or ESAM homozygous-KO (Homo) males and ESAM Het females (right: ♂, male; ♀, female). (B) The number of embryonic day (E) 14.5 fetuses obtained from ESAM Het males and ESAM Het females. (C) The crown rump length (CRL) of E14.5 fetuses obtained from ESAM Het males and ESAM Het females (WT, n = 24; Het, n = 35; Homo, n = 26). (D) The number of mononuclear cells (MNCs) in E14.5 fetal livers (WT, n = 24; Het, n = 35; Homo, n = 26). (E) The numbers of B220^+^ B cells, Gr1^+^ myeloid cells, and Ter119^+^ mature erythroid cells in E14.5 FLs (WT, n = 13; Het, n = 20; Homo, n = 23). (F) Numbers of Lin^−^ Sca1^+^ cKit^High^ (LSK), LSK CD48^−^, and LSK CD150^+^ CD48^−^ cells in E14.5 FLs (WT, n = 24; Het, n = 35; Homo, n = 34). Data are shown as means ± SEM. Statistically significant differences are represented by asterisks: ^∗^p < 0.05, ^∗∗^p < 0.01, ^∗∗∗^p < 0.001. See also Figure S1.

To evaluate how ESAM deficiency affected embryonic and infant mortality, genotype data from ESAM-deficient mice were collected. We performed genotyping when newly born pups were approximately 4 weeks old. By mating heterozygous-KO (Het) males and Het females, wild-type (WT) and Het mice were born and raised as estimated by the Mendelian ratio, whereas the number of homozygous-KO (Homo) mice was approximately 50% fewer than expected (Figure 1A, left). The same was true when Homo males and Het females were mated (Figure 1A, right). We did not detect high mortality of ESAM-null neonates after birth, suggesting that about a half of ESAM-null embryos may have a mortal disadvantage in the developmental process before and/or around birth. Surviving Homo ESAM-null mice were somewhat smaller than WT and Het littermates, as reported previously (Ishida et al., 2003), but the Homo pups grew up normally to become fertile.

### ESAM Deficiency Disrupted Hematological Development in the Fetal Liver

A previous study showed that ESAM deficiency caused no obvious malfunction in the development of the vascular system (Ishida et al., 2003). Similarly, we did not find any apparent abnormalities in the circulation system or other organogenesis of ESAM-null fetuses until embryonic 14.5 (E14.5). Therefore, we inferred that ESAM deficiency caused unfavorable events in the development of the hematopoietic system after mid-gestation because high ESAM expression marked definitive HSCs proliferating in the fetal liver (FL) (Yokota et al., 2009). In addition, ESAM-null HSCs from the adult BM did not immediately reconstitute hematopoiesis, particularly erythropoiesis, after 5-FU treatment, suggesting that ESAM expression played important roles in the proliferative phase of hematopoiesis (Sudo et al., 2012, Sudo et al., 2016).

To examine whether fetal hematopoiesis was affected by ESAM deficiency, we evaluated the FL, which is the main organ for hematopoiesis during mid-gestation, by comparing Homo ESAM-deficient fetuses and their WT or Het littermates. At E14.5, whereas no significant reduction was detected in the number and crown rump length of ESAM-null fetuses (Figures 1B and 1C), total mononuclear cell numbers in the livers were decreased by 20% compared with those of WT mice (Figure 1D). Flow cytometry experiments with lineage-related markers revealed that both B lymphoid and granulocyte lineage cells were decreased in Homo FLs (Figure 1E). These data suggested that development of multilineage hematopoiesis was influenced by ESAM deficiency. Therefore, we examined the population of hematopoietic stem/progenitor cells in ESAM-null FLs using Sca1, CD48, and CD150 antigens, which are commonly used to identify definitive HSCs. We observed marked reductions in HSCs and progenitors in Homo FLs. Indeed, the lineage marker-negative Sca1^+^ c-kit^Hi^ (LSK) fraction, which contained HSCs and multipotent progenitors, was decreased by approximately 50% in Homo FLs (Figure 1F, left). Similar results were observed for LSK CD48^–^ and LSK CD48^–^ CD150^+^ fractions, which showed more stringent criterion for HSC purification, and the numbers of HSCs were less than half of normal counts in many ESAM-null embryos (Figure 1F, middle and right).

### ESAM Deficiency Caused High Mortality in Fetuses after E15.5

**Figure 2:**
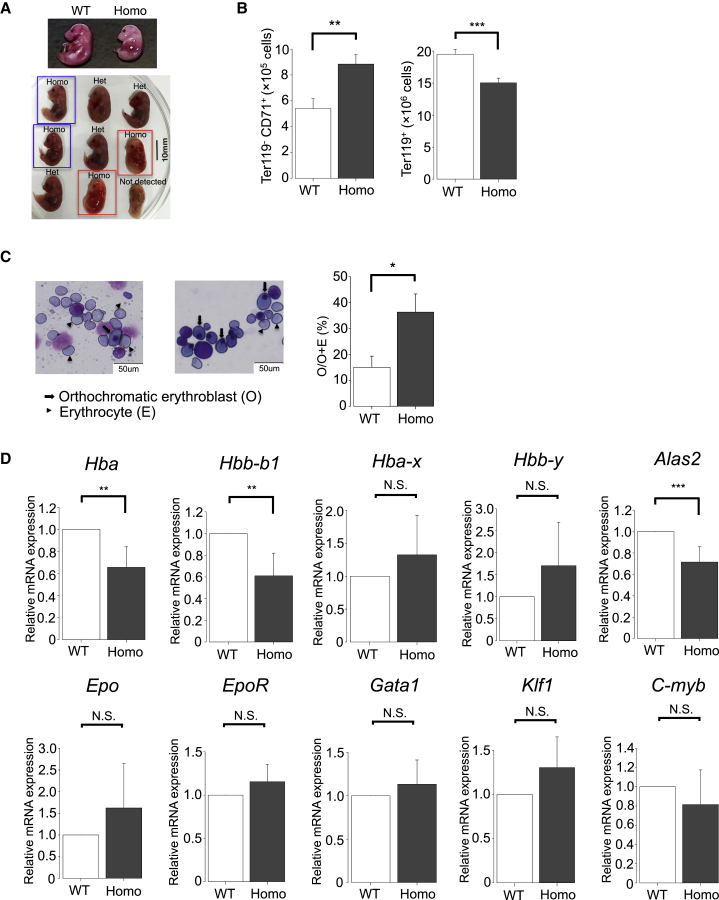
ESAM Deficiency Was Associated with High Mortality in Fetuses after E15.5 (A) Phenotypic comparison of ESAM Homo fetuses with their littermates after E15.5. Typical images of E17 (upper) and E17.5 (lower) fetuses are shown. Living and dead ESAM Homo fetuses are surrounded by blue and red squares, respectively. (B) Absolute number of Ter119^–^ CD71^+^ immature erythroblasts and Ter119^+^ mature erythroblasts/erythrocytes in FLs at E14.5 (n = 3, each group). (C) Representative smear specimens of FLs at E14.5 (left). Arrows indicate orthochromatic erythroblasts, and arrowheads indicate enucleated erythrocytes. The percentages of orthochromatic erythroblasts per orthochromatic erythroblasts plus enucleated erythrocytes are shown (right) (n = 3, each group). (D) Results of real-time qPCR analyses of E16.5 WT and ESAM Homo KO FLs (three independent experiments). Data are shown as means ± SEMs. Statistically significant differences are represented by asterisks: ^∗^p < 0.05, ^∗∗^p < 0.01, ^∗∗∗^p < 0.001.

The numbers and sizes of ESAM-null fetuses were comparable with those of WT and Het littermates at E14.5 (Figures 1B and 1C). Although we observed no clear differences in the appearances of fetuses among the three genotypes, there were some abnormalities in developing hematopoiesis (Figures 1B–1F), suggesting that ESAM deficiency may cause fatal events after mid-gestation. Thus, we sequentially examined the development of ESAM-null fetuses after E14.5. As a result, we found that some ESAM-null fetuses exhibited severe anemia after E15.5 and that approximately half of those anemic fetuses died before E17.5 (Figure 2A). Although we could not attribute fetal death exclusively to the anemia, we did not notice other lethal events, such as hemorrhage or maldevelopment, in the dying fetuses.

Next, we examined how erythropoiesis was affected in ESAM-null FLs at E14.5. Developmental abnormalities in erythropoiesis were already evident in FLs at E14.5. The number of mature Ter119^+^ erythroid cells was significantly decreased, whereas CD71^+^ Ter119^–^ immature erythroid cells were increased (Figure 2B), indicating the delay of erythroid cell differentiation. In addition, the frequency of nucleated orthochromatic erythroblasts was more conspicuous in E14.5 ESAM-deficient FLs than in WT FLs (Figure 2C).

We then performed real-time qPCR analyses to determine the expression of erythropoiesis-related genes in E16.5 ESAM-null FLs. The results revealed significant reduction in mRNA levels of adult globins (*Hba* and *Hbb-b1*), whereas the mRNA levels of embryonic globins were sustained (Figure 2D). In addition, transcripts for an erythroid-specific isoenzyme of 5-aminolevulinic acid synthase 2 (*Alas2*), the first and rate-limiting enzyme in the heme biosynthesis pathway, were also significantly reduced in ESAM-null FLs. In contrast, transcripts for erythropoietin (*Epo*), Epo-receptor (*EpoR*), and several transcription factors essential for erythropoiesis, such as *Gata1*, Krueppel factor 1 (*Klf1*), and *c-myb*, were not decreased by ESAM deletion. These results showed that development of adult-type hemoglobin synthesis was seriously impaired in ESAM-null fetuses.

### ESAM-Null HSCs Exhibited Functional Disruption of Differentiation in Culture

The findings above suggested that ESAM deficiency was related to the development of adult-type hematopoiesis. The number of HSCs with the definitive phenotype markedly decreased in ESAM-null FLs at E14.5 (Figure 1F), and the HSC reduction was detectable as early as E13.5 (Figure S1). However, life-threatening events occurred in hematopoiesis after E15.5, implying that the incapability of HSCs to produce mature blood cells was potentially lethal. Therefore, we tested the quality of ESAM-null HSCs using *in vitro* culture.

**Figure 3:**
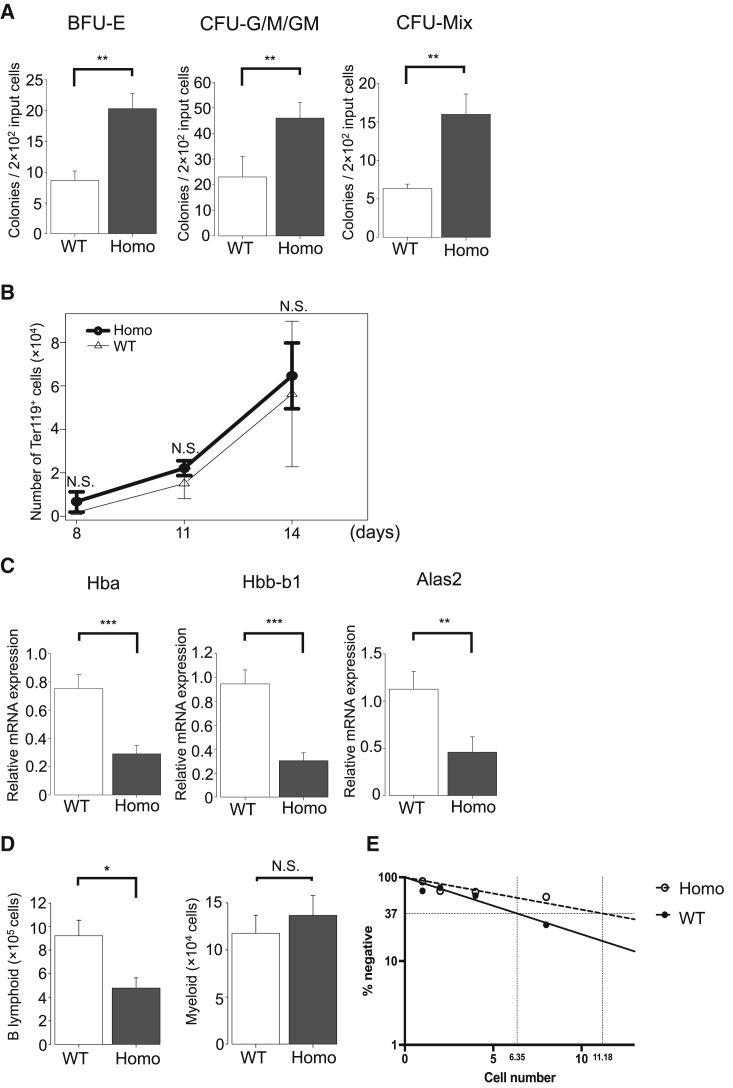
ESAM-Null HSCs Exhibited Functional Disruption of Differentiation in Culture (A) The sorted LSK CD48^–^ cells of E14.5 WT or ESAM Homo KO littermates were cultured in methylcellulose medium. The number of granulocyte colony-forming units (CFU-G), macrophage colony-forming units (CFU-M), granulocyte-macrophage colony-forming units (CFU-GM), erythroid burst-forming units (BFU-E), or mixed erythroid-myeloid colony-forming units (CFU-Mix) are shown (n = 3, each group). (B) The sorted LSK CD48^–^ cells of E14.5 WT or ESAM Homo KO littermates were cocultured in BM stromal cell lines (MS-5), under appropriate conditions to produce erythroid cells. After 8, 11, and 14 days of culture, cells were collected and analyzed by fluorescence-activated cell sorting (FACS). The numbers of Ter119^+^ erythroid cells are shown over time (n = 4, each group). (C) The mRNA expression levels of *Hba*, *Hbb-b1*, and *Alas2* in the BFU-E colonies analyzed by qRT-PCR (n = 15, each group). (D) Sorted LSK cells of E14.5 WT or ESAM Homo KO littermates (100 cells/well) were cocultured with MS-5 under conditions to produce B lymphoid and myeloid cells. After 10 days of culture, cells were collected and analyzed by FACS. The numbers of CD19^+^ B lymphoid cells and Mac1^+^ myeloid cells are shown (n = 4, each group). (E) FL LSK CD48^–^ HSCs collected at E14.5 from WT or ESAM Homo KO fetuses were subjected to limiting dilution analyses in the MS-5 coculture system. Input cell numbers corresponding to 37% negative value are shown in rectangles. Data are shown as means ± SEM. Statistically significant differences are represented by asterisks: ^∗^p < 0.05, ^∗∗^p < 0.01, ^∗∗∗^p < 0.001.

LSK CD48^–^ cells were sorted from E14.5 WT or ESAM-null FLs and were cultivated in methylcellulose medium containing stem cell factor (SCF), interleukin-3 (IL-3), IL-6, and EPO, which supported the clonal growth of myeloid-erythroid progenitors. Surprisingly, ESAM-null HSCs generated more myeloid-erythroid colonies than WT HSCs (Figure 3A). The sizes of the generated colonies were similarly large, suggesting that ESAM-null HSCs could proliferate and differentiate into mature myeloid-erythroid cells by responding to optimal cytokines. Similar results were obtained when HSCs were cocultured with a murine stromal cell line, MS-5, in the presence of SCF and EPO, which supported the growth of myeloid-erythroid lineage cells (Tokunaga et al., 2010). The numbers of Ter119^+^ erythroid cells produced from WT and ESAM-null HSCs were comparable over time (Figure 3B).

The data obtained in methylcellulose colony assays and the anemic phenotype of ESAM-null fetuses seemed to be contradictory. Based on gene expression data (Figure 2D), we assumed that, although ESAM-null HSCs could produce erythroid cells, their ability to synthesize adult-type hemoglobin may be impaired. To test this hypothesis, we examined the expression levels of adult-type hemoglobin-related genes in erythroid burst-forming units (BFU-E) colonies. Fifteen BFU-E colonies were individually picked up from WT and ESAM-null HSC cultures and were subjected to real-time qPCR. The results clearly showed that transcripts for *Hba*, *Hbb-b1*, and *Alas2* genes were markedly reduced in ESAM-null HSC-derived BFU-E colonies (Figure 3C).

Notably, lymphopoietic activity, which is an authentic feature of definitive HSCs, was impaired in ESAM-null HSCs. When HSCs were cocultured with MS-5 cells in the presence of SCF, FLT3-ligand, and IL-7, which supported the growth of B-lymphocytes and myeloid cells (Kouro et al., 2005), the output of CD19^+^ B cells from ESAM-null HSCs was significantly lower than that from WT cells, although myeloid cell growth was equivalent (Figure 3D). In addition, limiting dilution analyses showed that the frequencies of progenitors with lymphopoietic potential were decreased by approximately 40% in the LSK CD48^–^ fraction of ESAM-null FLs (Figure 3E).

### ESAM-Null FL HSCs Caused an Anemic Phenotype after *In Vivo* Transplantation

**Figure 4:**
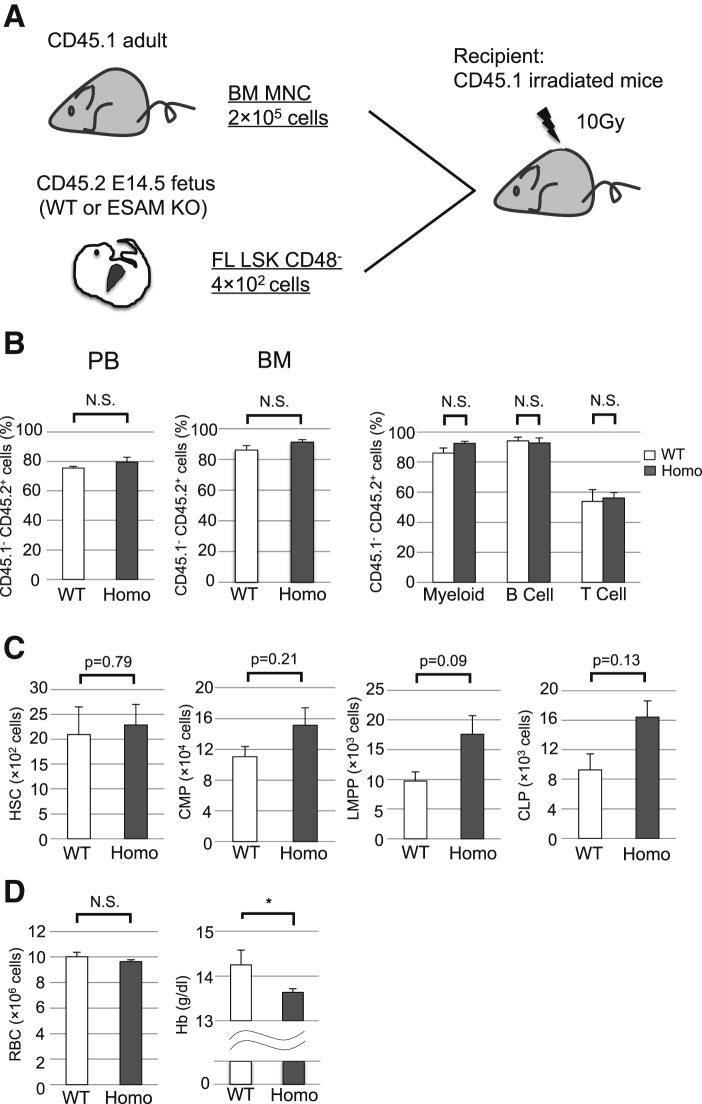
ESAM-Null FL HSCs Caused an Anemic Phenotype after *In Vivo* Transplantation Four hundred E14.5 FL LSK CD48^-^ cells from WT or ESAM Homo KO mice were transplanted into lethally irradiated CD45.1 recipient mice (WT, n = 4; Homo, n = 6). Fifteen weeks after transplantation, recipient mice were sacrificed and analyzed. (A) Schematic showing the transplantation protocol. (B) The percentages of CD45.1^–^ CD45.2^+^ donor-type chimerism in whole peripheral blood (PB, left) or bone marrow (BM, middle). Percentages of donor-type chimerism in Mac1^+^Gr1^+^ myeloid, B220^+^ B cell, and CD3^+^ T cell fractions (right). (C) The number of LSK CD150^+^ CD48^–^ HSCs, Lin^−^ c-Kit^+^ Sca-1^–^ IL-7Ra^−^ CD4^–^ CD8^–^ IgM^−^ CD34^+^ FCγR^low^ common myeloid progenitors (CMPs), LSK Flt3^+^ IL-7Ra^−^ lymphoid-primed multipotent progenitors (LMPPs), and Lin^−^ c-Kit^low^ Sca-1^–/low^ Flt3^+^ IL-7Ra^+^ common lymphoid progenitors (CLPs) in BM. (D) Peripheral blood cell counts of red blood cells (RBCs) and hemoglobin (Hb). Data are shown as means ± SEM. Statistically significant differences are represented by asterisks: ^∗^p < 0.05. See also Figure S2.

Next, we performed competitive repopulation assays to examine the differentiation potential of HSCs from ESAM-null FLs in adult mice. Four hundred LSK CD48^–^ HSCs sorted from CD45.2^+^ E14.5 ESAM-null or WT FLs were transplanted into lethally irradiated CD45.1^+^ congenic WT mice with 2 × 10^5^ CD45.1^+^ BM cells (Figure 4A). After 15 weeks, we determined the contribution levels of CD45.2^+^ cells to recipient hematopoiesis. Chimerism of CD45.2^+^ donor cells in mononuclear cells of peripheral blood (PB) or BM did not differ between the two groups (Figure 4B, left and middle). In addition, chimerism did not differ among lineages (Figure 4B, right). The numbers of CD45.2^+^ HSCs, common myeloid progenitors, lymphoid-primed multipotent progenitors, and common lymphoid progenitors were slightly higher in ESAM-null HSC-transplanted recipients, although these differences were not statistically significant (Figure 4C). These results suggested that the engraftment and proliferation abilities of HSCs and progenitor cells in WT adult BM were not disrupted by ESAM deficiency. In addition, hematopoietic colony units were more abundant in the recipient BM of ESAM-null HSC transplantation in comparison with that of WT HSCs (Figure S2). In contrast, hemoglobin levels in recipients were significantly lower in the PB of ESAM-null HSCs, despite the lack of difference in red blood cell counts (Figure 4D). Therefore, although ESAM expression was dispensable for the reconstitution of hematopoiesis in WT mice by HSCs from FLs, these results showed that ESAM expression played a role in maintaining adult-type hemoglobin synthesis ability.

### ESAM Deficiency Influenced Gene Expression Profiles in HSCs

**Figure 5:**
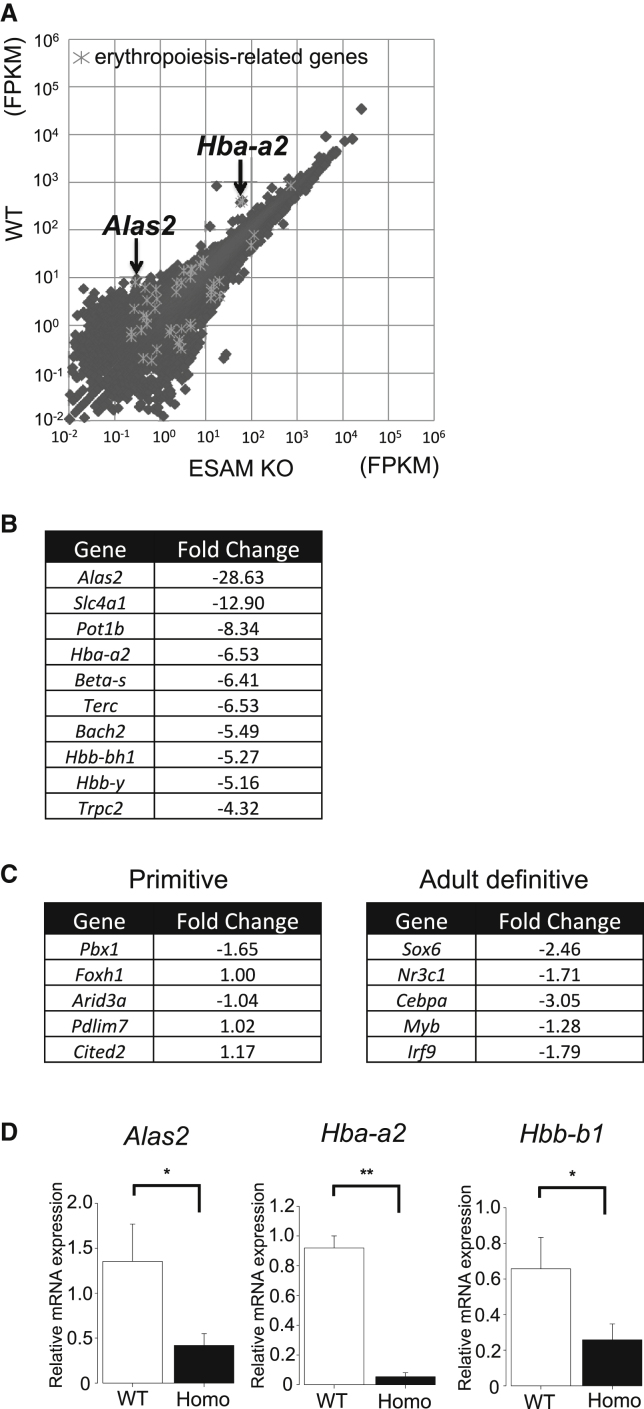
ESAM Deficiency Influenced Gene Expression Profiles in HSCs Gene expression profiles of E14.5 FL LSK CD48^–^ cells between WT and ESAM Homo KO fetuses were compared by RNA-seq analysis. (A) Scatterplots comparing transcript levels (in fragments per kilobase of exon per million fragments) in ESAM Homo KO (x axis) and WT (y axis) mice. (B) Top 10 “erythropoiesis-related” genes that were downregulated by ESAM deletion. Fold changes were calculated as the ratio of ESAM Homo to WT. (C) Fold change data of selected genes related to primitive and definitive erythropoiesis. (D) The mRNA expression levels of *Alas2*, *Hba-a2*, and *Hbb-b1* in LSK CD48^–^ HSCs confirmed by qRT-PCR (three independent experiments). Data are shown as means ± SEM. Statistically significant differences are represented by asterisks: ^∗^p < 0.05, ^∗∗^p < 0.01. See also Figure S3.

Next, we conducted RNA sequencing (RNA-seq) analysis to compare gene expression patterns of WT and ESAM-null HSCs to elucidate the molecular mechanisms involved in the developmental failure of hematopoiesis in ESAM-null FLs. The RNA-seq data demonstrated the downregulation of 2,559 genes and upregulation of 1,148 genes in E14.5 LSK CD48^–^ ESAM-null FL cells compared with the gene expression in WT cells (Figure 5A). We found a marked reduction in erythropoiesis-related gene expression (Figure 5B). Indeed, transcripts from not only adult-type but also embryonic-type globin genes, such as *Hbb-y* and *Hbb-bh1*, were dramatically decreased in ESAM-null HSCs. The reduction in *Alas2* gene expression was most obvious among erythropoiesis-related genes, and the extent of reduction was approximately by 30-fold less in ESAM-null HSCs (Figure 5B). In addition, although representative “primitive erythroid-enriched” genes were not affected, “definitive erythroid-enriched” genes (Kingsley et al., 2013) were downregulated in ESAM-null HSCs (Figure 5C). Real-time qPCR analyses confirmed the significant downregulation of *Alas2* and adult-type globin genes (*Hba-a2* and *Hbb-b1*; Figure 5D). These results supported our hypothesis that adult hemoglobin synthesis was impaired by ESAM deficiency and further suggested that the cause of fatal anemia was initiated as early as at the HSC stage. We also found that some B cell-related genes were impaired in ESAM-null HSCs (Figure S3A). Among these genes, *Notch2*, *Tnfaip3*, *Ahr*, and *Malt1* have been reported to be involved in the nuclear factor κB (NF-κB) pathway (Klein et al., 2015, Lee et al., 2000, Vogel and Matsumura, 2009, Zhang et al., 2014), which is essential for B cell development (Kaileh and Sen, 2012). Together with the results of culture experiments (Figures 3D and 3E), these findings suggested that ESAM may be related to B cell production via the NF-κB pathway. In addition, several HSC-related genes were upregulated, presumably to compensate for the deletion of ESAM (Figure S3B). Among them, IL-27 was greatly upregulated (Figures S3B and S3C). The increase in colony-forming progenitors (Figures 3A and S2) may be at least partially due to the upregulation of autocrine secreted IL-27 in ESAM-deficient HSCs because IL-27 is known to stimulate the proliferation of HSCs and contribute to myeloid lineage differentiation synergistically with SCF *in vitro* (Seita et al., 2008).

### ESAM Directly Influenced the Expression of Erythropoiesis-Related Genes in HSCs

**Figure 6:**
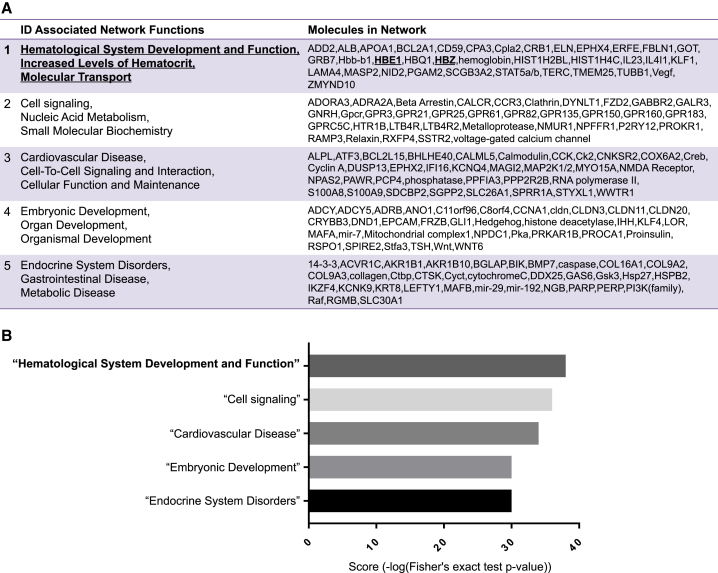
ESAM Expression on HSCs Directly Influenced Their Quality E14.5 WT FL-derived LSK CD48^–^ cells were either untreated (ESAM minus) or incubated with a rat monoclonal antibody against mouse ESAM (ESAM plus). The extracted RNA samples were used to conduct RNA-seq and bioinformatic analyses by Ingenuity Pathway Analysis (IPA). (A) Top 5 networks affected in this experiment are shown. (B) The IPA network scores of each network described in (A) are shown in a horizontal bar graph. Scores indicate the negative exponent of the p value which is based on the hypergeometric distribution and is calculated with the Fisher's exact test. See also Figure S4.

Because ESAM mediates cell-cell interactions through homophilic binding, we hypothesized that ESAM expressed on HSCs may transduce some signals that affect gene expression profiles, particularly erythropoiesis-related genes. To test this hypothesis, we used an antibody crosslinking method (Hegen et al., 1997, Murray and Robbins, 1998). As a result, we found that the crosslinking of ESAM with an anti-ESAM antibody affected various genes in HSCs. Expression of 365 genes was upregulated, whereas that of 358 genes was downregulated (Figure S4A). The ingenuity pathway network analysis indicated that hematological system development and function, increased levels of hematocrit, and molecular transport networks, which were associated with hemoglobin synthesis, showed the greatest changes (Figures 6A and 6B). Indeed, two globin genes (*HBE1* [*Hbb-bh1*] and *HBZ* [*Hba-x*]) were upregulated. In addition, we observed that *Gdf15*, which promotes hemoglobin synthesis by suppressing hepcidin secretion (Tanno et al., 2007), was the most strongly induced gene following ESAM crosslinking (Figure S4B). We also found that several genes related to Rho GTPase, which has been reported to be activated by ESAM on ECs (Wegmann et al., 2006) and is closely related to erythropoiesis (Kalfa and Zheng, 2014), were upregulated (Figure S4C). These results suggested that ESAM expressed on HSCs was directly involved in the regulation of erythropoiesis, particularly for hemoglobin-related genes.

### ESAM Expressed on ECs Was Critically Involved in Developing Hematopoiesis

**Figure 7:**
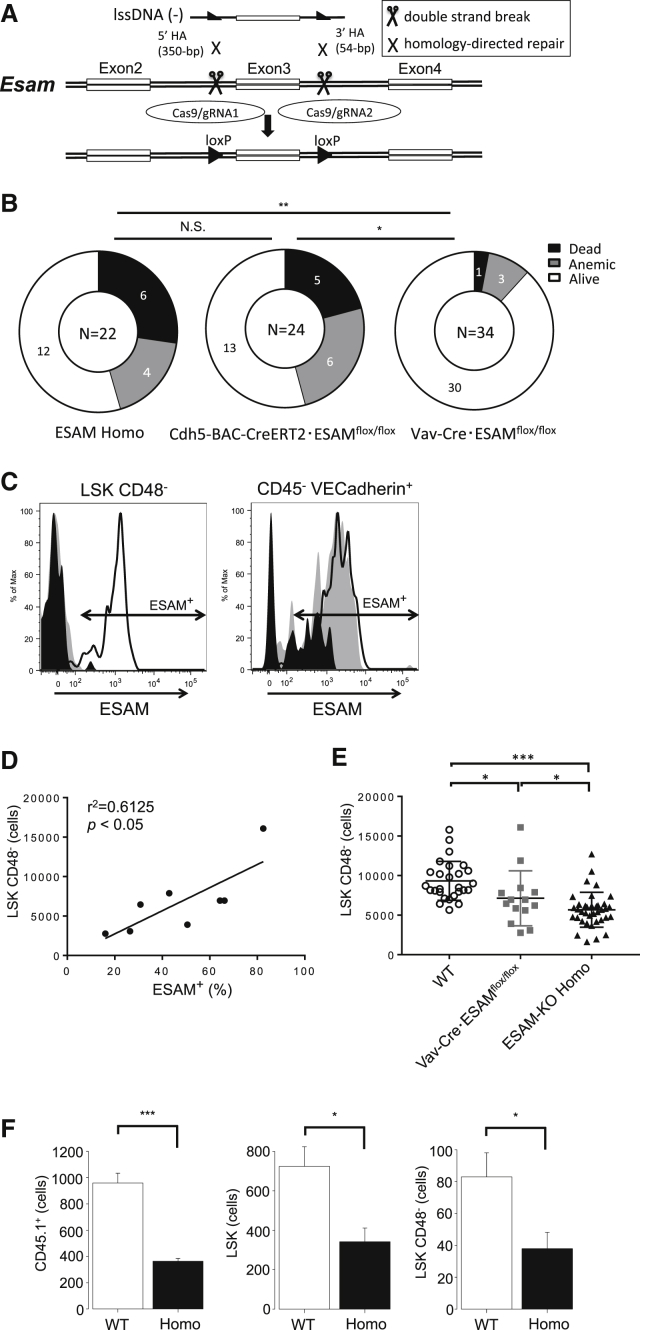
ESAM on Endothelial Cells Were Essential for Developing Hematopoiesis (A) Schematic approach to generate ESAM-floxed alleles using the CRISPR/Cas system with long single-stranded DNA (lssDNA) composed of the targeted exon flanked by two *loxP* sites. (B) Pie charts showing the numbers of “Dead,” “Anemic,” or “Alive” fetuses analyzed between E16.5 and E17.5 (ESAM Homo, n = 22; Cdh5-BAC-CreERT2·ESAM^flox/flox^, n = 24; Vav-Cre·ESAM^flox/flox^, n = 34). We were unable to determine the genotype of one of the six Dead fetuses included in the ESAM Homo group because of severe damage to its tissues. The difference between each group was analyzed by Fisher's exact test. (C) Representative histograms showing the expression levels of ESAM on LSK CD48^-^ HSCs (left) and CD45^–^ VE-cadherin^+^ ECs (right) in E14.5 FLs in Vav-Cre·ESAM^wt/wt^ (solid line) and Vav-Cre·ESAM^flox/flox^ littermates (light gray or dark gray). ESAM expression on ECs was deleted with different efficiency even in the Vav-Cre·ESAM^flox/flox^ littermates (light gray or dark gray). (D) The results of Pearson's correlation analysis between the percentages of ESAM^+^ cells in CD45^–^ VE-cadherin^+^ ECs (x axis) and the number of LSK CD48^-^ HSCs (y axis) in E14.5 Vav-Cre·ESAM^flox/flox^ FLs are shown (right, n = 8). (E) The numbers of LSK CD48^−^ cells in E14.5 FLs (WT, n = 27; Vav-Cre·ESAM^flox/flox^; n = 14; ESAM Homo, n = 37). (F) The absolute numbers of CD45.1^+^ cells, LSK cells, and LSK CD48^–^ HSCs after 4 days of organ culture with DMEM/10% FCS are shown (n = 4, each group). Data are shown as means ± SEM. Statistically significant differences are represented by asterisks: ^∗^p < 0.05, ^∗∗^p < 0.01 ^∗∗∗^p < 0.001. See also Figure S5.

The results above demonstrated that ESAM expressed on HSCs played important roles in developing erythropoiesis in the liver. However, it was still unclear whether ESAM expression on HSCs was essential or whether that on another lineage, i.e., ECs, was also involved. Accordingly, we generated ESAM-flox mice using the CRISPR with long single-stranded DNA (lssDNA) inducing conditional KO alleles (CLICK) method, which was recently developed by our group (Miyasaka et al., 2018) (Figure 7A). Using this model, we first attempted to establish endothelial lineage-specific ESAM-cKO mice by crossing ESAM-flox mice with Cre-ERT2-expressing mice under control of the VE-cadherin gene promoter (Okabe et al., 2014). After peritoneal administration of tamoxifen to pregnant females on E12.5, E14.5, or E15.5, the FLs of their fetuses were examined on E17.5 (Figure S5A). Analyses of these fetuses showed that, as was the case in conventional ESAM-null mice, severe anemia occurred in approximately half of Cdh5-BAC-CreERT2·ESAM^flox/flox^ fetuses (Figure 7B). However, we noticed that ESAM expression was almost completely deleted in both ECs and HSCs in this model (Figures S5C and S5D), even with single tamoxifen administration on E15.5.

Then, we exploited another ESAM-cKO model obtained by crossing ESAM-flox mice with Cre-recombinase-expressing mice under the control of the *Vav* gene promoter (Figure S5B). Although this Vav-Cre model had been widely used as a hematopoietic cell-specific gene KO system, we knew that the EC lineage was also affected with mouse-to-mouse variations (Joseph et al., 2013). Indeed, we observed that ESAM expression on ECs was deleted with diverse efficiency, whereas that on HSCs was completely absent (Figures 7C and S5E). We determined that hemoglobin concentrations in circulating blood were significantly decreased in the Vav-Cre·ESAM^flox/flox^ fetuses (Figure S5F); surprisingly, however, the anemic phenotypic and fetal mortality of those fetuses were less critical (Figure 7B, right). Notably, the absolute number of LSK CD48^–^ HSCs in FLs was positively correlated with the remaining ESAM expression levels on ECs (Figure 7D). Furthermore, the number of HSCs was decreased in Vav-Cre·ESAM^flox/flox^ FLs but still retained in comparison with ESAM-KO Homo FLs (Figure 7E). These results suggested that ESAM expressed on ECs also play some roles in the development of definitive HSCs and erythropoiesis.

To elucidate the significance of ESAM expression of endothelial lineage on the development of HSCs more directly, we conducted FL-reaggregated organ culture experiments in which WT LSK CD48^–^ HSCs from E14.5 FLs of CD45.1^+^ mice were cultured with ESAM^+^ or ESAM^−^ ECs. The reaggregates containing ESAM^−^ ECs produced fewer LSK CD48^–^ HSCs, LSK HSPCs, and total CD45.1^+^ hematopoietic cells than those containing ESAM^+^ ECs (Figure 7F). These results suggested that endothelial ESAM was important for the maintenance and proliferation of HSCs.

## Discussion

The development of hematopoiesis during the embryonic and fetal periods is composed of multiple waves that arise in diverse organs (Dzierzak and Speck, 2008, Mikkola and Orkin, 2006). Although HSCs and self-renewing multipotent progenitors are responsible for lifelong blood production (Busch et al., 2015, Sun et al., 2014), that is not the case in embryos, where blood cells emerge in accordance with developmental requirements. Authentic HSCs emerge from the hemogenic endothelium around mid-gestation and maintain definitive hematopoiesis throughout late fetal stages and adult life. These HSCs, as well as the hemogenic endothelium, express high amounts of ESAM on the cell surface (Yokota et al., 2009); however, the functional significance of ESAM in developing hematopoiesis has yet to be determined. In this study, we clearly showed that ESAM plays important roles in the development of definitive hematopoiesis, particularly of adult-type erythropoiesis.

Among developing hematopoietic cells, erythroid cells are indispensable for supporting the rapid growth of multiple organs in embryos and fetuses. Indeed, the first murine erythroid cells emerge in the yolk sac as “primitive” erythropoiesis around E7.5 (Palis et al., 1999, Silver and Palis, 1997), which is 3 days earlier than the emergence of HSCs (Medvinsky and Dzierzak, 1996). After generation in the yolk sac, the erythroid lineage develops sequentially with three waves, which differ in origin, cell morphology, and gene expression patterns (McGrath and Palis, 2008). The first and second waves are derived from committed progenitors in the yolk sac, which mature or expand in the blood stream or FL (Kingsley et al., 2004, Kingsley et al., 2006, Lux et al., 2008). The third wave, arising from definitive HSCs, gradually supersedes the yolk sac-derived erythropoiesis after mid-gestation. Although we observed that high mortality in ESAM-deficient fetuses was mainly due to the developmental failure of the erythroid lineage, the lethal influence of ESAM deletion was observed after E15.5. Thus, the timing of death of ESAM-deficient fetuses supported the interpretation that ESAM expression is critical in the development of definitive, but not primitive erythropoiesis. The reason why not all the ESAM-null fetuses died would be explained by the heterogeneity of definitive HSCs, which might have buffered the life-threatening anemia. A recent paper demonstrated that, among mouse KO lines causing intrauterine lethality, more than two-thirds of cases are associated with placental malformation (Perez-Garcia et al., 2018). However, we believe this was not the case in our study because ESAM-deficient fetuses grew normally until E15.5 without placental dysplasia.

Although various endothelial lineage-related antigens have been reported to mark developing HSCs, few are known to have critical functions in the development of hematopoiesis. Tie2/angiopoietin receptor-2 and its ligand angiopoietin-1 are involved in the maintenance of HSCs in adult BM; however, they seem to be dispensable for the generation and differentiation of definitive HSCs in fetuses (Arai et al., 2004, Puri and Bernstein, 2003, Zhou et al., 2015). Among endothelial-related makers, CD31/PECAM-1 belongs to the immunoglobulin superfamily and is known to share a similar function with ESAM in terms of leukocyte migration through VE junctions (Muller, 1993, Vaporciyan et al., 1993, Wegmann et al., 2006). In contrast, in CD31/PECAM-1-deficient mice, the number of HSCs was expanded along with excessive megakaryopoiesis, and the phenotype substantially differed from that of ESAM-deficient mice (Wu et al., 2007). Thus, to the best of our knowledge, ESAM is a unique endothelial-related antigen that has been shown to play roles in the development and function of definitive HSCs.

Importantly, although ESAM-deficient HSCs in FLs retained long-term reconstituting ability and the robust capability for erythroid cell production, the synthesis of adult-type globins was significantly disrupted. This abnormality was more evident when HSC clones were cultured in methylcellulose medium containing sufficient amounts of cytokines. The expression of *Alas2*, encoding an essential enzyme for heme biosynthesis, was also markedly decreased in BFU-E colonies derived from ESAM-deficient HSCs. These observations clearly showed that ESAM deficiency deprived HSCs of authentic qualities required for adult globin production. Notably, downregulation of *Alas2* and adult-type globin genes was obvious as early as in HSCs. Thus, during the developmental process, ESAM-mediated interactions may be indispensable for the acquisition of authentic differentiation potential in arising HSCs. The observed impairment of lymphopoietic activity in ESAM-deficient HSCs also supported this interpretation.

We speculated that ESAM deficiency may affect HSC quality through indirect rather than direct mechanisms because, unlike CD31/PECAM-1, ESAM structurally lacks the immunoreceptor tyrosine-based activating or inhibitory motif. Thus, it was surprising that the expression of more than 700 genes was affected by short-term crosslinking with ESAM. Although crosslinking with antibodies does not necessarily recapitulate the physiological interaction via ESAM, our analysis demonstrated that the gene network associated with hemoglobin synthesis was most influenced in HSCs. Interestingly, embryonic-type globin genes, including *Hba-x* and *Hbb-bh1*, were upregulated by ESAM crosslinking. This result was consistent with the RNA-seq data of ESAM-deficient HSCs, in which transcripts for both adult and embryonic globin genes were reduced. These findings are not contradictory to our claim for the particular function of ESAM in adult-type globin synthesis because embryonic and adult-type globin genes are sequentially located and share common *cis*-elements as the locus control region (LCR) (Stamatoyannopoulos, 2005). Because the LCR is open and active, even in HSCs and multipotent progenitors (Jimenez et al., 1992, Papayannopoulou et al., 2000), ESAM may transduce some signals to regulate the LCR as early as the HSC stage. In addition, the globin switch to the adult-type is mediated by chromatin looping and repressive transcription factors during the maturation of fetuses (Kingsley et al., 2006, Masuda et al., 2016, Noordermeer and de Laat, 2008, Palis, 2008), which is likely promoted by ESAM-mediated interactions in an indirect manner. Further studies are needed to explore the molecular mechanisms involved in these functions.

Our original conditional KO system has shed light on the importance of ESAM expression in the endothelial lineage. Since exclusive gene targeting in the endothelial lineage was nearly impossible at this stage, even with the Cdh5-BAC-CreERT2 system (Okabe et al., 2014), we tried to circumvent this by using a Vav-cre-induced conditional KO system. Although the number of HSCs in the Vav-cre-induced ESAM-cKO FL was comparable with that in the WT FL when the ESAM expression level was sustained in the endothelial lineage, HSCs decreased in parallel with the decrease in ESAM-expressing ECs. Furthermore, the anemic phenotype was less severe, and fetal mortality was less evident in Vav-cre-induced ESAM-cKO fetuses. The results shown by organ culture indicated the contribution of endothelial ESAM to the proliferation and maintenance of HSCs. These results were unexpected because we previously observed that the contribution of ESAM expression on nonhematopoietic cells to HSC maintenance was subtle in adult BM of transplantation-induced chimeric mice (Sudo et al., 2016). However, this contradiction may be explained in terms of the difference between the FL and adult BM. Indeed, various types of cells have been reported to serve as the so-called “HSC niche” in adult BM (Morrison and Scadden, 2014), whereas most HSCs are located adjacent to sinusoidal ECs in the FL (Iwasaki et al., 2010). We previously showed the intimate association between ESAM^Hi^ HSCs and ECs in adult BM after 5-FU administration (Sudo et al., 2012), implying that ECs also play important roles in expanding hematopoietic cells in the FL. Because ESAM expression on ECs in the developing liver compensated for ESAM deficiency on HSCs to ameliorate the lethal anemic phenotype, endothelial ESAM may interact with undetermined molecules on developing HSCs, the functions of which may overlap with those of ESAM. We are currently investigating this important issue.

In conclusion, our data strongly suggested that ESAM expression on ECs and HSCs played important roles in the development of definitive hematopoiesis. In particular, declined adult-type hemoglobin synthesis due to ESAM deficiency was found to be critically involved in intrauterine lethality after mid-gestation. Mutations in *Esam* may be related to rare congenital anemia because *Esam* deficiency markedly reduced the expression of *Alas2* in HSCs, mutations in which are known to cause hereditary sideroblastic anemia. Furthermore, our observations provided a theoretical background for potential clinical applications of artificial ESAM-mediated interactions to mitigate prolonged anemia after BM injury or in patients with thalassemia.

## Experimental Procedures

### Mice

ESAM-KO mice were provided by Dr. Ishida (Ishida et al., 2003). Vav-iCre transgenic mice were purchased from Jackson Laboratory. Although Vav-iCre^+^ females are recommended for mating in this model (Joseph et al., 2013), we also mated Vav-iCre^+^ males with ESAM-flox mice and used their progeny in this study because no apparent difference was observed in our experiments. ESAM-flox mice were generated using the CLICK method (Miyasaka et al., 2018). First, we designed two gRNAs and lssDNA. gRNAs targeted introns on either side of exon 3 in the *Esam* gene, and lssDNA contained exon 3 flanked by two *loxP* sequences with a 54-bp 3′ homology arm (HA) and an extended 350-bp 5′ HA. Two gRNAs, IssDNA, and *Cas9* mRNA were transferred into C57BL/6JJcl embryos (CLEA Japan). After electroporation, the embryos were cultured and transferred into the oviducts of pseudo-pregnant mice on day 0.5 (Jcl: ICR, CLEA Japan). All animal studies were approved by the institutional review board of Osaka University (permit no. 25-098-002). The day of vaginal plug observation was considered as E0.5.

## Author Contributions

T.U. and T.Y. designed the experiments and analyzed the data. Experiments were performed by T.U., T.Y., D.O., Y.U., T.M., T.S., and T.I. with assistance from Y.Kubota, Y.S., Y.D., T.O., and R.N. The manuscript was written by T.U., T.Y., D.O., Y.U., T.M., and Y.Kanakura with assistance from A.T., M.I., S.E., H.S., and K.O.

## Supplemental Information

Document S1. Supplemental Experimental Procedures, Figures S1–S5, and Tables S1 and S2

Document S2. Article plus Supplemental Information
