# Supplementary material for: Endothelial Cell-Selective Adhesion Molecule Contributes to the Development of Definitive Hematopoiesis in the Fetal Liver
Source: Stem Cell Reports. 2019 Dec 5;13(6):992–1005. doi: 10.1016/j.stemcr.2019.11.002 (PMC6915804; doi:10.1016/j.stemcr.2019.11.002)
Supplement: Document S1. Supplemental Experimental Procedures, Figures S1–S5, and Tables S1 and S2 [file mmc1.pdf]

Stem Cell Reports, Volume *13*

Supplemental Information

Endothelial Cell-Selective Adhesion Molecule Contributes to the Development of Definitive Hematopoiesis in the Fetal Liver

Tomoaki Ueda, Takafumi Yokota, Daisuke Okuzaki, Yoshihiro Uno, Tomoji Mashimo, Yoshiaki Kubota, Takao Sudo, Tomohiko Ishibashi, Yasuhiro Shingai, Yukiko Doi, Takayuki Ozawa, Ritsuko Nakai, Akira Tanimura, Michiko Ichii, Sachiko Ezoe, Hirohiko Shibayama, Kenji Oritani, and Yuzuru Kanakura

Figure S1

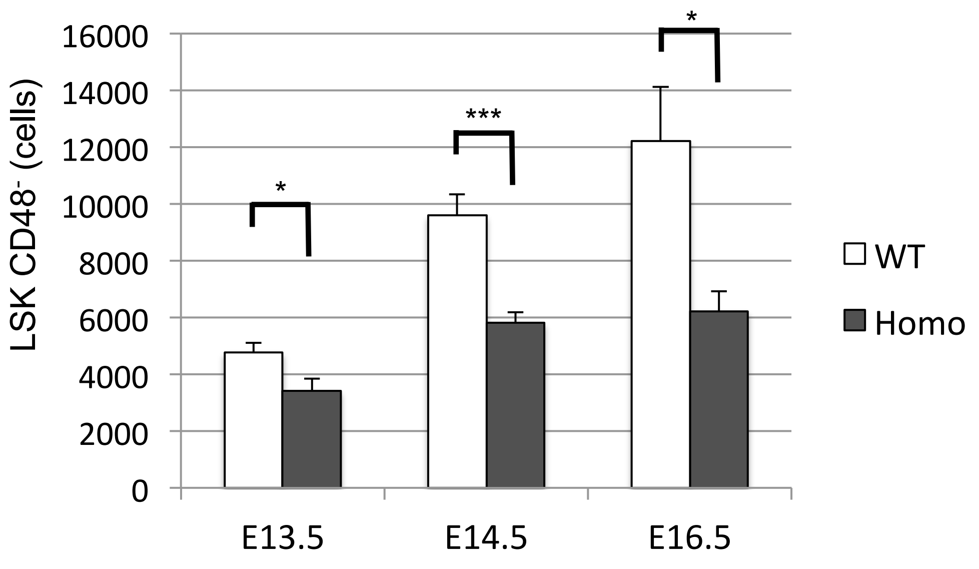

Figure S1. The number of HSCs decreased in ESAM-null FLs, Related to Figure 1F.
The number of LSK CD48$^-$ HSCs in fetal livers on embryonic days (E) 13.5 (WT, n = 9; Homo, n = 6) , 14.5 (WT, n = 16 ; Homo, n = 24) , and 16.5 (WT, n = 5 ; Homo, n = 6). Data are shown as means ± SEMs. Statistically significant differences are represented by asterisks (*p < 0.05, ***p < 0.001).

Figure S2

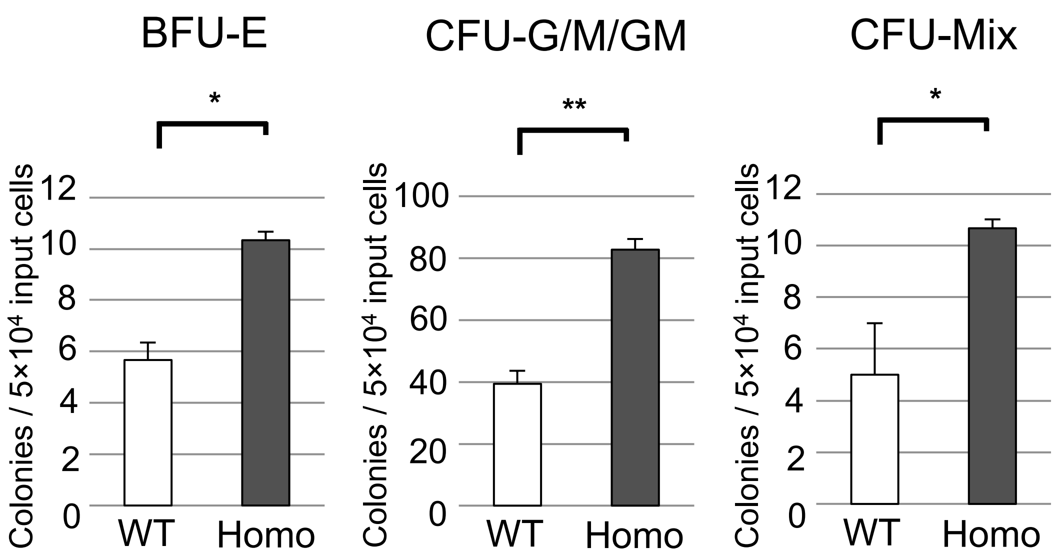

Figure S2. ESAM-null HSC derived BM cells produced larger number of colonies, Related to Figure 4.
The number of colonies produced from 5.0×10^4 unfractionated BM cells obtained from ESAM Homo KO or WT FL HSCs transplanted mice (n = 3, each group). Data are shown as means ± SEMs. Statistically significant differences are represented by asterisks (*$p < 0.05$, **$p < 0.01$).

Figure S3

A

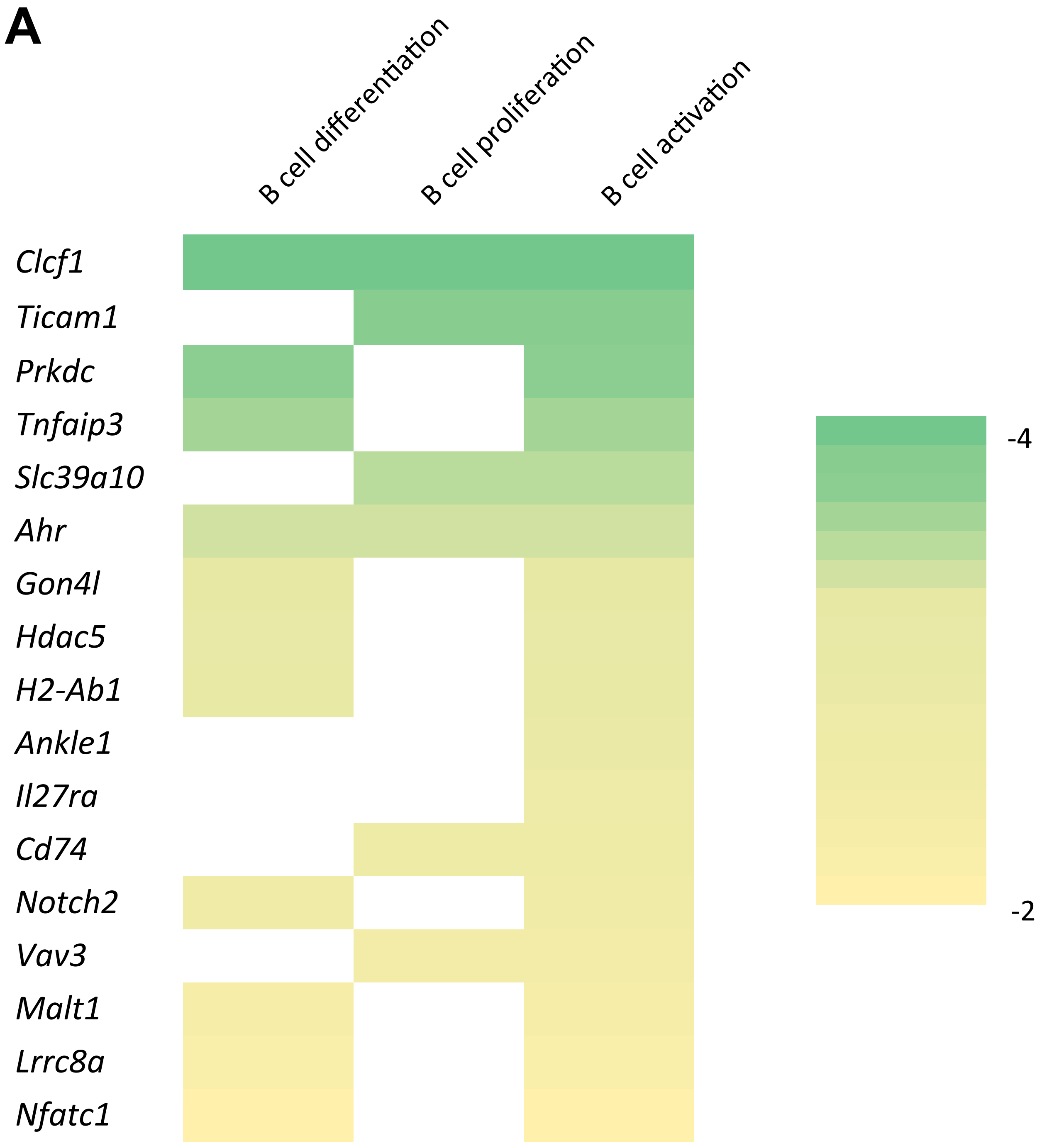

B

Gene	Fold Change
Sema4a	5.82
IL-27	5.72
Sfrp2	5.38
Tgfb2	4.41
Hes1	4.32

C

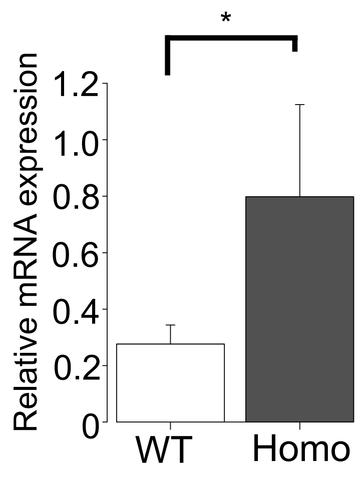

Figure S3. ESAM deficiency influenced B cell-related and HSC-related gene expression, Related to Figure 5.
Gene expression profiles in the E14.5 fetal liver LSK CD48$^-$ fraction from ESAM Homo KO and WT mice analyzed by RNA-seq. (A) Heat maps of selected genes related to “B cell differentiation”, “B cell proliferation”, and “B cell activation” are shown (fold change < -2). Fold change was calculated as the ratio of ESAM Homo to WT. (B) Top five “HSC-related” genes upregulated by ESAM deletion. (C) The mRNA expression levels of *IL-27* in LSK CD48$^-$ cells of E14.5 WT or ESAM Homo KO littermates (WT, n = 5; Homo, n = 3). Data are shown as means ± SEMs. Statistically significant differences are represented by asterisks (*$p < 0.05$).

Figure S4

A

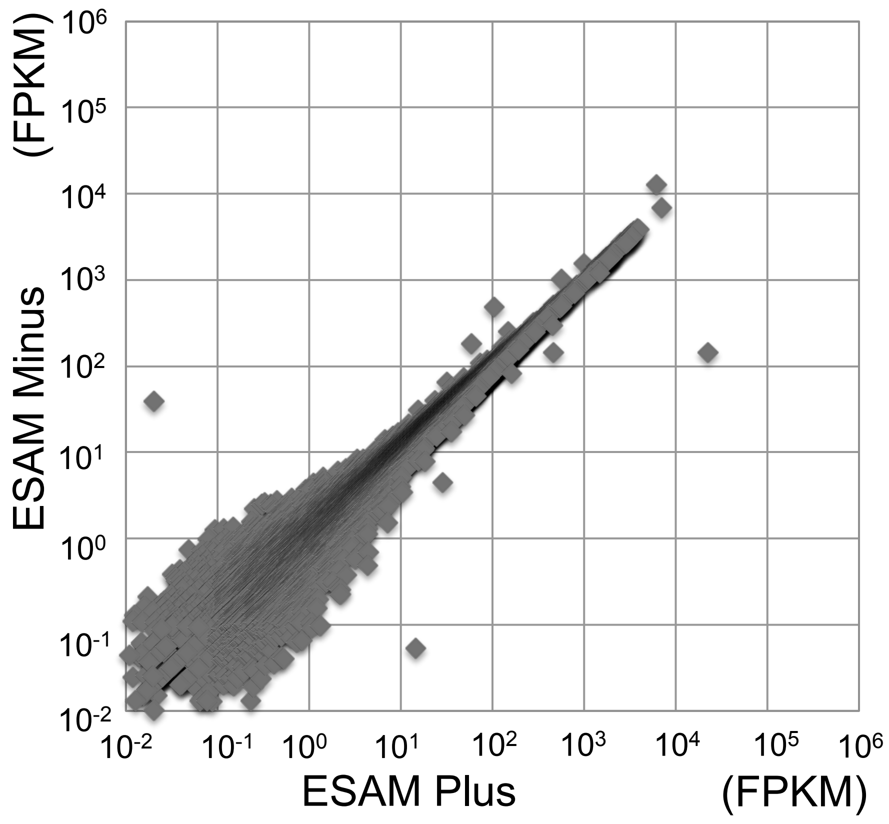

B

Gene	Fold Change
Gdf15	11.23
Ctrl	9.19
4930592A05Rik	8.67
Figf	8.17
Grifin	8.17

C

Gene	Fold Change
Cnksr1	2.81
Arhgap24	2.55
Arhgap44	2.18
Rhod	2.11
Tubb1	2.04

Figure S4. ESAM crosslinking affected gene expression of HSCs, Related to Figure 6.
E14.5 WT fetal liver-derived LSK CD48$^-$ cells were either untreated (ESAM minus) or incubated with a rat monoclonal antibody against mouse ESAM (ESAM plus). (A) Scatterplots comparing transcript levels (in fragments per kilobase of exon per million fragments) in ESAM plus (x-axis) and ESAM minus (y-axis). (B) Top five genes upregulated by ESAM crosslinking. (C) Top five upregulated genes related to Rho GTPases. Fold change was calculated as the ratio of ESAM plus to ESAM minus.

Figure S5

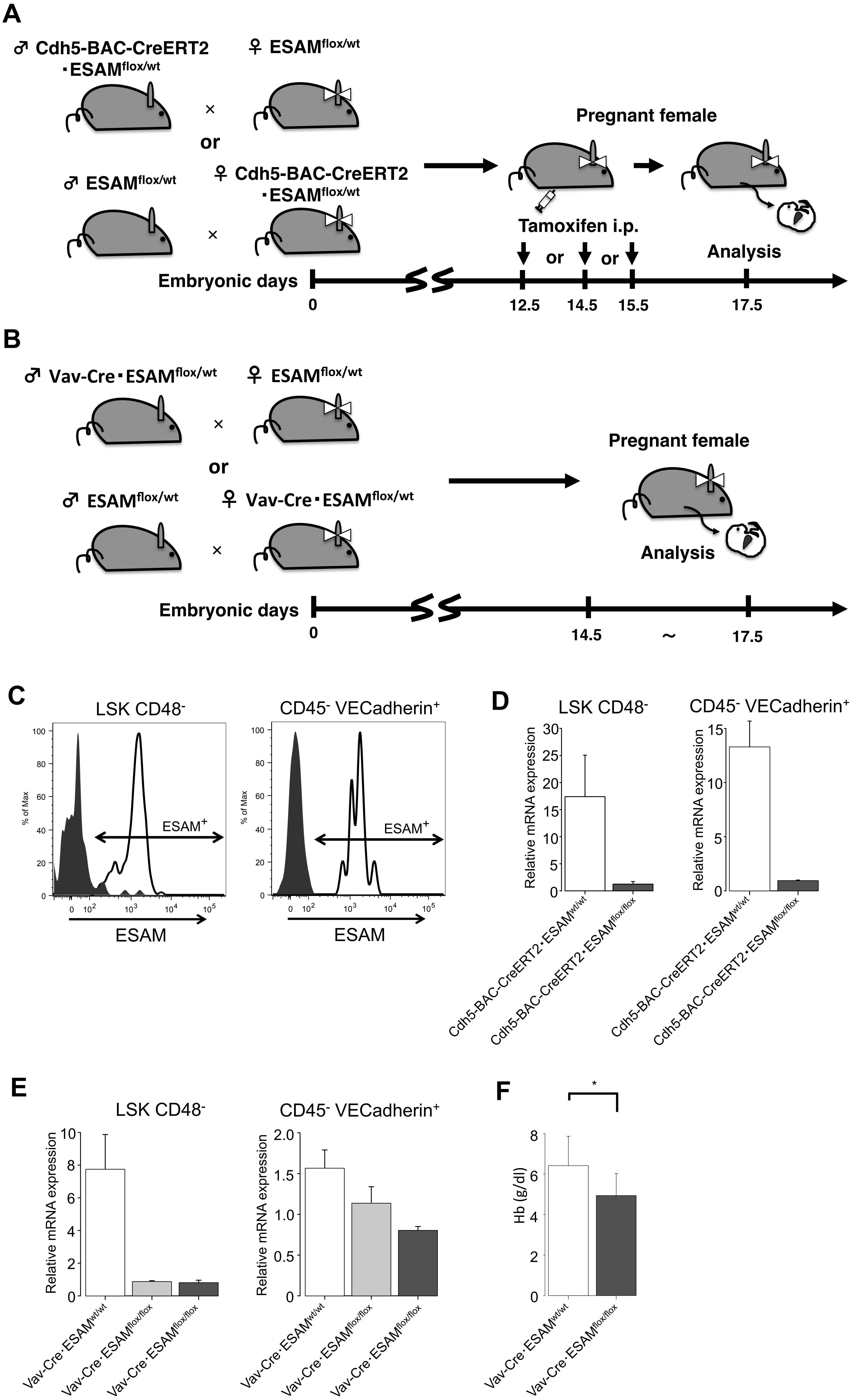

Figure S5. Scheme of the experimental procedure and the additional results using ESAM-flox mice, Related to Figure 7

Cdh5-BAC-CreERT2·ESAM$^{flox/wt}$ male mice (♂) were crossed with ESAM$^{flox/wt}$ female (♀) mice or ESAM$^{flox/wt}$ male mice were crossed with Cdh5-BAC-CreERT2·ESAM$^{flox/wt}$ female mice. Tamoxifen (10-1000 ug) was administered intraperitoneally for pregnant mice on embryonic day (E) 12.5, 14.5, or 15.5, and their fetuses were analyzed on E17.5. (B) Vav-Cre·ESAM$^{flox/wt}$ male mice were crossed with ESAM$^{flox/wt}$ female mice or ESAM$^{flox/wt}$ male mice were crossed with Vav-Cre·ESAM$^{flox/wt}$ female mice. Their fetuses were analyzed between E14.5 and 17.5. (C) Representative histograms showing ESAM expression on LSK CD48$^-$ HSCs (left) and CD45$^-$ VE-cadherin$^+$ endothelial cells (ECs) (right) in E17.5 FLs of Cdh5-BAC-CreERT2·ESAM$^{wt/wt}$ (solid line) and Cdh5-BAC-CreERT2·ESAM$^{flox/flox}$ littermates (dark gray). (D) The mRNA expression levels of *Esam* in LSK CD48$^-$ HSCs (left) and CD45$^-$ VE-cadherin$^+$ ECs of E17.5 FLs of Cdh5-BAC-CreERT2·ESAM$^{wt/wt}$ and Cdh5-BAC-CreERT2·ESAM$^{flox/flox}$ littermates (3 independent experiments). (E) The mRNA expression levels of *Esam* in LSK CD48$^-$ HSCs (left) and CD45$^-$ VE-cadherin$^+$ ECs (right) of E14.5 FLs of Vav-Cre·ESAM$^{wt/wt}$ (open bar) and Vav-Cre·ESAM$^{flox/flox}$ littermates (light gray bar or dark gray bar) (3 independent experiments). (F) Peripheral blood hemoglobin (Hb) levels in E17.5 fetuses (Vav-Cre·ESAM$^{wt/wt}$; n = 7, Vav-Cre·ESAM$^{flox/flox}$; n = 8). Data are shown as means ± SEMs. Statistically significant differences are represented by asterisks (*$p < 0.05$).

Table S1. Antibodies used in this study, Related to Figure 1-4, 7, S1, and S5.

Antibodies	Source	Identifier
Purified anti-mouse CD16/32 antibody, Clone 93	Biolegend	Cat# 100308; RRID:AB_312801
Purified Rat anti-mouse ESAM antibody, Clone 1GB	eBioscience	Cat# 14-5852
FITC anti-mouse/human CD11b antibody, Clone M1/70	Biolegend	Cat# 101206; RRID:AB_312789
FITC anti-mouse CD34 antibody, Clone	eBioscience	Cat# 11-0341-82
FITC anti-mouse CD45 antibody, Clone 30-F11	Biolegend	Cat# 103108; RRID:AB_312973
FITC anti-mouse CD45.1 antibody, Clone A20	Biolegend	Cat# 110706; RRID:AB_313495
FITC anti-mouse CD45.2 antibody, Clone 104	Biolegend	Cat# 109806; RRID:AB_313443
FITC anti-mouse CD48 antibody, Clone HM48-1	Biolegend	Cat# 103404; RRID:AB_313019
FITC anti-mouse Ly-6G/Ly-6C (Gr-1) antibody, Clone RB6-8C5	Biolegend	Cat# 108406; RRID:AB_313371
FITC anti-mouse TER119/erythroid cell antibody, Clone TER119	Biolegend	Cat# 116206; RRID:AB_313707
PE anti-mouse CD3ε antibody, Clone 145-2C11	Biolegend	Cat# 100308; RRID:AB_312673
PE anti-mouse CD45.1 antibody, Clone A20	Biolegend	Cat# 110708; RRID:AB_110708
PE rat anti-mouse CD71 antibody, Clone C2	BD Biosciences	Cat# 553267
PE anti-mouse CD150 (SLAM) antibody, Clone TC15-12F12.2	Biolegend	Cat# 115904; RRID:AB_313683
PE anti-mouse ESAM antibody, Clone 1GB/ESAM	Biolegend	Cat# 136203; RRID:AB_1953300
Biotin anti-mouse CD8a antibody, Clone 53-6.7	BD Biosciences	Cat# 553029
Biotin anti-mouse CD45.2 antibody, Clone 104	Biolegend	Cat# 109804; RRID:AB_313441
Biotin anti-mouse CD127 (IL-7Ra) antibody, Clone SB/199	Biolegend	Cat# 121103; RRID:AB_493501
Biotin anti-mouse CD150 (SLAM) antibody, Clone TC15-12F12.2	Biolegend	Cat# 115907; RRID:AB_345277
Biotin anti-mouse IgM antibody, Clone RMM-1	Biolegend	Cat# 406504; RRID:AB_315054
PerCP-Cy™5.5 rat anti-mouse CD3 molecular complex	BD Biosciences	Cat# 560527
PerCP/Cy5.5 anti-mouse CD4, Clone GK 1.5	Biolegend	Cat# 100434; RRID:AB_893324
PerCP-Cy™5.5 rat anti-mouse CD45R/B220 antibody, Clone RA3-6B2	BD Biosciences	Cat# 553093
PerCP/Cy5.5 anti-mouse Ly-6A/E (Sca-1) antibody, Clone D7	Biolegend	Cat# 108124; RRID:AB_893615
PerCP/Cy5.5 anti-mouse Ly-6G/Ly-6C (Gr-1) antibody, Clone RB6-8C5	Biolegend	Cat# 108426; RRID:AB_893557
PerCP/Cy5.5 anti-mouse TER-119/erythroid cell antibody, Clone TER119	Biolegend	Cat# 116226; RRID:AB_893635
PE/Cy7 anti-mouse/human CD11b antibody, Clone M1/70	Biolegend	Cat# 101216; RRID:AB_312799
PE/Cy7 anti-mouse/human CD45R/B220 antibody, Clone RA3-6B2	Biolegend	Cat# 103222; RRID:AB_313005
PE/Cy7 anti-mouse CD117 (c-Kit) antibody, Clone 2B8	Biolegend	Cat# 105814; RRID:AB_313223
PE/Cy7 anti-mouse Ly-6A/E (Sca-1) antibody, Clone D7	Biolegend	Cat# 108114; RRID:AB_493596
PE/Cy7 anti-mouse Ly-6G/Ly-6C (Gr-1) antibody, Clone RB6-8C5	Biolegend	Cat# 108416; RRID:AB_313381
APC anti-mouse CD3ε antibody, Clone 145-2C11	Biolegend	Cat# 100312; RRID:AB_312677
APC anti-mouse CD16/32 antibody, Clone 93	eBioscience	Cat# 17-0161-81
APC anti-mouse/human CD45R/B220 antibody, Clone RA3-6B2	Biolegend	Cat# 103212; RRID:AB_312997
APC anti-mouse CD45.1 antibody, Clone A20	Biolegend	Cat# 110714; RRID:AB_313503
APC anti-mouse CD117 (c-Kit) antibody, Clone 2B8	Biolegend	Cat# 105812; RRID:AB_313221
APC anti-mouse CD135 antibody, Clone A2F10	Biolegend	Cat# 135310; RRID:AB_2107050
APC anti-mouse CD144 (VE-cadherin) antibody, Clone BV13	Biolegend	Cat# 138011; RRID:AB_10679039
APC anti-mouse ESAM antibody, Clone 1GB/ESAM	Biolegend	Cat# 136207; RRID:AB_2101658
APC/Cy7 anti-mouse/human CD11b antibody, Clone M1/70	Biolegend	Cat# 101226; RRID:AB_830642
APC/Cy7 anti-mouse CD45 antibody, Clone 30-F11	Biolegend	Cat# 103115; RRID:AB_312980
APC/Cy7 anti-mouse CD45.2 antibody, Clone 104	Biolegend	Cat# 109824; RRID:AB_830789
APC/Cy7 anti-mouse Ly-6A/E (Sca-1) antibody, Clone D7	Biolegend	Cat# 108126; RRID:AB_10645327
APC/Cy7 anti-mouse Ly-6G/Ly-6C (Gr-1) antibody, Clone	Biolegend	Cat# 108424; RRID:AB_2137485
Brilliant Violet 421™ anti-mouse CD48 antibody, Clone HM48-1	Biolegend	Cat# 103427; RRID:AB_10895922
PE-CF594 streptavidin	BD Horizon	Cat# 562318
Anti-Rat IgG MicroBeads	Miltenyi Biotech	Cat# 130-048-501

Table S2. Primers used in this study, Related to Figure 2D, 3C, 5D, S3C, S5D, and S5E.

Gene	Forward	Reverse
Alas2	5′-TGTCCCAGCCACATCATCCCC-3′	5′-GCGCAGTAGCTCCTCACCACG-3′
β-Actin	5′-GCGTGACATTAAAGAGAAGCTG-3′	5′-CTCAGGAGGAGCAATGATCTTG-3′
C-myb	5′-AAGACCCTGAGAAGGAAAAGCG-3′	5′-GTGTTGGTAATGCCTGCTGTCC-3′
Epo	5′-CCACCCTGCTGCTTTTACTC-3′	5′-CTCAGTCTGGGACCTTCTGC-3′
EpoR	5′-CCCAAGTTTGAGAGCAAAGC-3′	5′-TGCAGGCTACATGACTTTCG-3′
Esam	5′-TGCCCACATTCTAGACCTCCA-3′	5′-CTCCTTTTGTCCTTTGACCCAG-3′
Gata1	5′-ACCACTACAACACTCTGGCG-3′	5′-CAAGAACTGAGTGGGGCGAT-3′
Hba	5′-CTCTCTGGGGAAGACAAAAGCAAC-3′	5′-GGTGGCTAGCCAAGGTCACCAGCA-3′
Hba-a2	5′-GGCCATGGTGGTGAATATGGCGAG-3′	5′-GCCTTGACCTGGGCAGAGCCGGGG-3′
Hba-x	5′-CTGTCTGCTGGTCACAATGG-3′	5′-GGGAGGAGAGGGATCATAGC-3′
Hbb-bh1	5′-TGGACAACCTCAAGGAGACC-3′	5′-TGCCAGTGTACTGGAATGGA-3′
Hbb-y	5′-CTTGGGTAATGTGCTGGTGA-3′	5′-GTGCAGAAAGGAGGCATAGC-3′
IL-27	5′-CACCTCCGCTTTCAGGTGC-3′	5′-AGGTATAGAGCAGCTGGGGC-3′
Klf1	5′-CAGCTGAGACTGTCTTACCC-3′	5′-AATCCTGCGTCTCCTCAGAC-3′

SUPPLEMENTAL EXPERIMENTAL PROCEDURES

Flow cytometry and cell sorting

Cells were blocked with an anti-CD16/32 (clone 93) antibody (BioLegend), incubated with various antibodies, and resuspended in 7-AAD-containing buffer. As a negative control, we used isotype-matched antibodies in flow cytometry experiments.
Flow cytometric analysis and sorting were performed using FACSCanto or FACSAriaIIu cytometers (BD Biosciences). Data analyses were performed with FlowJo software (Tree Star). Antibodies used in this study are shown in Table S1.

Methylcellulose culture and qRT-PCR

We suspended 600 sorted LSK CD48$^-$ cells from ESAM KO or WT FLs in 3 mL Methocult GF M3434 (StemCell Technologies), distributed the cells into three 35-mm dishes, and incubated the cells in 5% CO_2 at 37°C. After 8 days of culture, colonies were counted and classified as granulocyte colony-forming units, macrophage colony-forming units, granulocyte-macrophage colony-forming units, BFU-E, or mixed erythroid-myeloid colony-forming units according to the shape and color under an inverted microscope. After counting, BFU-E colonies were picked up and suspended in 500 µL TRIzol. Similarly, we suspended 1.5×10^5 unfractionated BM cells obtained from ESAM KO or WT FL HSCs transplanted mice in 3 mL Methocult GF M3434. Colonies were analyzed after 8 days of culture.

MS-5 stromal cell coculture

MS-5 stromal cells were prepared at a concentration of 3×10^4 cells/well in 24-well tissue culture plates (1 day) before the seeding of sorted cells. Cells were cultured in α-minimum essential medium (α-MEM; Invitrogen, Carlsbad, CA, USA) supplemented with 10% fetal calf serum (FCS), rm SCF (10 ng/mL), rm Flt3-ligand (20 ng/mL), rm IL-7 (1 ng/mL), 2-mercaptoethanol (50 µM), and DuP-697 (1 µM) to produce B or myeloid cells and cultured in α-MEM supplemented with 10% FCS, rm SCF (50 ng/mL), and hEPO (150 ng/mL) to produce erythroid cells. The cultures were fed every 3 or 4 days by removing half of the medium and replacing it with fresh medium. Cells were then maintained for 14 days. Cytokines were added fresh each time the medium was replaced.

FL-reaggregated organ culture

Five thousand Lin$^-$ CD45$^-$ VE-Cadherin$^+$ ECs were sorted from ESAM KO or WT E14.5 FLs and mixed with 3.5×10^2 LSK CD48$^-$ HSCs from C57BL/6-Ly5.1 (CD45.1) E14.5 FLs. To support hematopoietic cell growth, 3×10^4 Lin$^-$ CD45$^-$ VE-Cadherin$^-$ hepatic parenchymal/stroma cells, which were originally negative for ESAM expression, were also added. Mixed cells were centrifuged, reaggregated to form a pellet, and cultured in DMEM/10% FCS.

RT-PCR analysis

Total RNA was extracted using a PureLink RNA Mini Kit (Thermo Fisher Scientific), with DNase treatment. A High Capacity RNA-to-cDNA Kit (Thermo Fisher Scientific) was used for cDNA synthesis. qRT-PCR was performed using an ABI PRISM 7900 HT (Applied Biosystems Inc., Foster City, CA, USA). Expression levels were normalized to the expression of the internal reference β-actin. Primers used in this study are shown in Table S2.

Competitive repopulation assay

Ly5 congenic mice were used for competitive repopulation assays. Four hundred LSK CD48$^-$ cells sorted from E14.5 ESAM Homo KO or WT FLs were mixed with 2×10^5 unfractionated adult BM cells obtained from C57BL/6-Ly5.1 (CD45.1) mice and transplanted into C57BL/6-Ly5.1 mice irradiated at a dose of 10 Gy. Fifteen weeks after transplantation, all recipients were sacrificed, and PB and BM cells were collected. The number of PB cells was counted using Sysmex KX-21 (Sysmex Corporation, Kobe).

Crosslinking analysis

LSK CD48$^-$ cells from E14.5 healthy FLs, on which a high amount of ESAM was uniformly expressed (Yokota et al., 2009), were incubated with or without rat monoclonal antibodies against mouse ESAM. Then, the cells were incubated with goat anti-rat IgG antibody-conjugated microbeads for 4 h and were subsequently applied to RNA-seq analyses.

RNA-seq analysis

For E14.5 LSK CD48$^-$ ESAM-null and WT FL cells, library preparation was performed using a TruSeq stranded mRNA sample prep kit (Illumina, San Diego, CA, USA) according to the manufacturer's instructions. For E14.5 WT fetal liver-derived LSK CD48$^-$ cells incubated with anti-ESAM monoclonal antibodies, cDNA was generated using a Clontech SMART-Seq Ultra Low Input RNA Kit (Takara Clontech, Mountain View, CA, USA). cDNA samples were sheared (200–500 bp) using a Covaris S220 (Covaris, Woburn, MA, USA) and prepared using KAPA Library Preparation Kits (Kapa Biosystems, Wilmington, MA, USA) for 75-bp single-end reads, according to the manufacturer's instructions. Sequencing was performed on an Illumina HiSeq 2500 platform in a 75-base single-end mode. Illumina Casava1.8.2 software used for base calling. Sequenced reads were mapped to the mouse reference genome sequences (mm10) using TopHat v2.0.13 in combination with Bowtie2 ver. 2.2.3 and SAMtools ver. 0.1.19. The fragments per kilobase of exon per million mapped fragments were calculated using Cufflinks ver.2.2.1. The raw data have been deposited in the NCBI Gene Expression Omnibus database: GSE116898.

Statistical analysis

Student's t-tests were used to compare data between two groups. Statistical analyses were conducted using error bars to represent standard errors of the means. Statistical analyses were performed using EZR (Saitama Medical Center, Jichi Medical University, Saitama, Japan) (Kanda, 2013), BellCurve for Excel (Social Survey Research Information), and GraphPad Prism7 (MDF). Results with p values of less than 0.05 were considered statistically significant.

SUPPLEMENTAL REFERENCES

Kanda, Y. (2013). Investigation of the freely available easy-to-use software 'EZR' for medical statistics. Bone Marrow Transplant *48*, 452-458.

Yokota, T., Oritani, K., Butz, S., Kokame, K., Kincade, P.W., Miyata, T., Vestweber, D., and Kanakura, Y. (2009). The endothelial antigen ESAM marks primitive hematopoietic progenitors throughout life in mice. Blood *113*, 2914-2923.
